# Evaluation of the mortality registry in Ecuador (2001–2013) – social and geographical inequalities in completeness and quality

**DOI:** 10.1186/s12963-019-0183-y

**Published:** 2019-03-28

**Authors:** Andrés Peralta, Joan Benach, Carme Borrell, Verónica Espinel-Flores, Lucinda Cash-Gibson, Bernardo L. Queiroz, Marc Marí-Dell’Olmo

**Affiliations:** 1 Agència de Salut Pública de Barcelona, Plaça Lesseps 1, 08023 Barcelona, Catalonia Spain; 2 Health Inequalities Research Group, Employment Conditions Knowledge Network (GREDS-EMCONET), Department of Political and Social Sciences, Universitat Pompeu Fabra, Ramon Trias Fargas, 25-27, 08005 Barcelona, Spain; 3 Johns Hopkins University, Pompeu Fabra University Public Policy Center, Barcelona, Spain; 4 Transdisciplinary Research Group on Socioecological Transitions (GinTRANS2), Universidad Autónoma Madrid, 28049 Madrid, Spain; 5 CIBER Epidemiología y Salud Pública (CIBERESP), Madrid, Spain; 6 Institut d’Investigació Biomèdica (IIB Sant Pau), Barcelona, Spain; 7 Department of Experimental and Health Sciences, Universitat Pompeu Fabra, Barcelona, Catalonia Spain; 8 Department of Demography and Cedeplar, Faculdade de Ciências Econômicas, FACE/UFMG, Campus Pampulha, Av. Antônio Carlos, 6627, Pampulha, Belo Horizonte, MG CEP 31270-901 Brazil

**Keywords:** Mortality, Mortality registries, Vital statistics, Health inequalities, Geographical inequalities

## Abstract

**Background:**

Mortality registries are an essential data source for public health surveillance and for planning and evaluating public policy. Nevertheless, there are still large inequalities in the completeness and quality of mortality registries between and within countries. In Ecuador, there have been few nationwide evaluations of the mortality registry and no evaluations of inequalities between provinces. This kind of analysis is fundamental for strengthening the vital statistics system.

**Methods:**

Ecological study assessing the completeness, quality and internal consistency of mortality data in the provinces of Ecuador, using 13 years of mortality data (2001–2013). *Completeness* was assessed using three types of death distribution methods (DDMs), *quality* by estimating the percentages of garbage codes and deaths with unspecified age or sex in the registered deaths, and *internal consistency* by estimating the percentage of deaths with reported causes of deaths considered impossible in some age–sex combinations. Finally, we propose a classification of the mortality registry in the studied areas based on completeness and quality.

**Results:**

Completeness estimates (mean of the three methods used) in the provinces ranged from 21 to 87% in women and from 35 to 89% in men. The percentage of garbage codes in the provinces ranged from 21 to 56% in women and from 25 to 52% in men. Garbage coding was higher in women and in older age groups. The percentage of deaths with unspecified age or sex, and the percentage of deaths with reported causes of deaths considered impossible in some age–sex combinations was low in all the studied areas. The mortality registry could only be classified as acceptable in one area for men and one area for women.

**Conclusions:**

We found substantial inequalities by sex, geographical areas and age in the completeness and quality of the mortality registry of Ecuador. The findings of this study will be helpful to direct measures to improve Ecuador’s vital statistics system and to generate strategies to reduce bias when using mortality data to analyse health inequalities in the country.

**Electronic supplementary material:**

The online version of this article (10.1186/s12963-019-0183-y) contains supplementary material, which is available to authorized users.

## Background

Civil registration and vital statistics systems, and particularly mortality registries, are essential components of public health surveillance systems in all countries. An adequate vital statistics system not only allows governments and academia to monitor and analyse the health of a population, but can also be a fundamental tool for the planning and evaluation of public policy [1]. Timely and correct vital statistics not only allow people to claim and exercise their basic human rights but could also be useful to inform and empower the population [2]. Moreover, evidence suggests that countries with better vital statistics systems have better health outcomes (even after consideration of income and other socioeconomic factors) [3].

In many countries, especially high-income ones, mortality registers are usually considered one of the most reliable sources for timely and accurate health statistics. The situation in many low- and middle-income countries is quite different. The 2015 Lancet Series “Counting Births and Deaths” reported disappointing global progress with civil registration and vital statistics systems [4]. In 2007, only 31 of the 192 member states of the World Health Organisation (13% of the world’s population) had mortality statistics with more than 90% completeness, using a recent version of the international statistical classification of diseases and related health problems (ICD8, ICD9 or ICD10), and with 10% or less of ill-defined codes [5]. Moreover, striking inequalities between countries and regions are seen in death registration. In a study analysing mortality data from all WHO member states, Mathers and colleagues reported three interesting issues: first, coverage of death registration ranged from 100% in the WHO Europe Region to less than 10% in the WHO Africa Region; second, completeness ranged from 100% in most high-income countries, to less than 50% in the Dominican Republic, Haiti, Lebanon and Morocco; and third, the percentage of deaths coded with ill-defined codes ranged from 4% in New Zealand to more than 40% in Thailand and Sri Lanka [6].

Inequalities in mortality statistics are not only seen between countries but also within countries, even though studies evaluating within-country differences are still uncommon. In Latin America, for example, studies in Brazil and Chile have found wide differences in the completeness and quality of mortality statistics between sub regions in each country [7, 8]. These types of study are necessary for two main reasons: 1) They help authorities to invest more efforts and resources to strengthen the vital statistics system in areas with the greatest deficiencies; and 2) They allow researchers and public health workers to use the available data, adjusting it for completeness and deficiencies in quality.

Some studies (considering completeness and ill-defined codes used among other indicators) have classified the mortality data in Ecuador as being of either low or medium performance [4, 6]. In addition, a study by Phillips and colleagues (see Additional file 3: Figure S1) suggests that the quality of mortality statistics in the country – and specially completeness – fell between the 1980s and 2010 [9]. In the latest edition of the Pan American Health Organization “Core Indicators – Health Situation in the Americas”, a completeness of 75% was reported at the national level; 9% of the registered deaths were assigned to ill-defined codes, and more than 16% of deaths were “assigned to causes not considered useful for public health purposes” (garbage codes) [10]. No studies analysing geographical inequalities in the death registration system have been carried out in Ecuador.

The objective of this study was to evaluate the completeness, quality and internal consistency of Ecuador’s mortality registry from 2001 to 2013, describing geographical inequalities (between provinces - first administrative level), and social inequalities (by age and sex) within the country.

## Methods

In this is ecological study, we collected mortality data from 2001 to 2013 for our analyses (762,131 deaths). Completeness and quality indicators were estimated at the national and provincial level. Ecuador has 24 provinces that can be located geographically in four regions: 1) The Galápagos Islands in the Pacific Ocean; 2) The coastal region in the western part of the continental territory; 3) The Amazonic region in the eastern portion of the continental territory; and 4) the Andean region, between the former two. During the study period, Santo Domingo and Santa Elena provinces were created in Ecuador. Santo Domingo was part of Pichincha and Santa Elena part of Guayas (the two most populated provinces in Ecuador). A stable cartography with 22 provinces was used (joining Pichincha with Santo Domingo, and Guayas with Santa Elena) to compare indicators between areas. At the time of the 2010 census, Ecuador had almost 14.5 million people. The population of the studied areas ranged from almost 4 million in Guayas/Santa Elena to less than 25,000 in Galapagos (details in Table 1).

A) Information sources

**Table 1:** Provincial and national completeness estimates (2001–2010)

Area	Women											Men				
	Population 2001	Population 2010	Deaths IC Period	Age Range Used	Population 2001	Population 2010	Deaths IC Period	GGB^a^	Age Range Used	SEG^b^	GGB - SEG^c^	Mean	GGB ^a^	SEG ^b^	GGB - SEG ^c^	Mean
Azuay	319,974	375,027	12,730	98.82	80.50	66.91	80.03	15–50	279,339	335,739	14,465	107.10	91.49	72.69	88.17	15–50
Bolivar	86,727	93,767	4280	98.81	71.91	71.29	78.83	20–60	83,969	89,975	5100	95.56	79.52	74.28	82.18	25–60
Cañar	111,707	119,621	4187	99.87	57.49	61.89	68.86	20–55	94,639	104,812	5137	95.54	79.06	70.87	80.59	15–50
Carchi	76,891	83,257	3147	80.20	56.76	55.98	62.56	15–50	75,106	80,905	3800	85.18	60.80	62.58	67.92	15–50
Cotopaxi	180,050	209,723	8369	99.92	83.67	72.23	83.79	20–55	169,507	197,990	10,264	101.66	84.40	65.66	81.27	15–50
Chimborazo	213,297	239,339	10,896	96.01	78.20	74.17	81.78	20–55	191,150	219,221	12,414	95.61	93.55	78.81	88.66	20–55
El Oro	258,108	295,256	8020	76.86	55.03	50.83	58.99	15–50	265,561	302,735	12,037	68.33	62.60	55.16	61.55	15–50
Esmeraldas	188,213	262,143	5758	43.07	86.01	63.62	59.33	15–50	197,331	270,912	9675	43.94	106.46	72.29	65.24	15–50
Guayas / Santa Elena	1,654,813	1,975,927	57,031	57.04	57.70	46.62	53.27	15–50	1,643,493	1,968,672	79,288	54.37	67.21	51.12	56.79	15–50
Imbabura	176,064	204,094	8243	101.18	86.67	73.53	85.66	15–55	167,689	193,105	9597	100.32	86.91	74.17	85.82	20–60
Loja	207,913	228,704	8141	100.81	62.41	59.31	70.09	15–50	198,316	221,638	9962	101.55	79.32	71.79	82.45	15–50
Los Rios	315,609	379,883	12,055	75.27	68.77	52.78	64.14	15–50	335,909	398,252	19,140	70.13	77.06	57.89	67.40	15–50
Manabi	592,524	681,575	20,872	82.71	63.62	55.61	65.51	15–50	599,521	689,525	29,639	85.19	74.86	61.20	72.40	15–50
Morona Santiago	57,891	73,126	1327	46.48	39.80	33.43	39.19	15–50	56,904	74,529	1806	54.03	63.17	49.05	54.82	15–50
Napo	38,776	50,641	1029	62.22	67.23	53.19	60.31	15–50	39,965	52,220	1577	71.98	88.74	64.05	73.57	15–50
Pastaza	29,686	41,394	718	40.59	73.71	56.18	53.57	15–50	31,808	42,084	1053	45.81	61.87	48.02	51.01	15–50
Pichincha / Santo Domingo	1,218,916	1,504,023	42,824	72.07	81.98	65.03	72.37	20–55	1,167,281	1,441,529	52,093	64.46	81.18	63.62	68.89	15–50
Tungurahua	227,308	258,907	10,747	98.69	83.24	82.22	87.44	15–55	213,470	244,014	12,569	99.48	87.86	78.87	87.94	15–55
Zamora Chinchipe	37,017	43,780	976	53.53	45.88	39.20	45.46	15–50	39,462	46,627	1391	47.16	52.06	41.78	46.62	15–50
Galapagos	8137	11,392	123	12.58	35.10	30.43	21.30	15–50	9618	12,238	187	25.46	45.66	38.83	34.51	15–50
Sucumbíos	58,774	83,431	1279	22.00	49.52	41.57	33.45	15–50	67,901	91,050	2589	28.53	58.70	46.24	40.70	15–50
Orellana	39,478	64,034	1073	28.85	105.65	74.30	52.10	15–50	45,147	70,655	1766	36.30	126.62	79.87	62.54	15–50
Ecuador	*6,132,183*	*7,293,907*	*224,300*	*71.97*	*67.67*	*57.21*	*65.00*	*15–50*	*6,010,246*	*7,165,170*	*296,474*	*71.76*	*74.68*	*60.09*	*68.23*	*15–50*

^a^ Generalized growth balance method

^b^ Synthetic extinct generations method

^c^ Hybrid generalized growth balance and synthetic extinct generations method

To estimate completeness, we used Ecuador’s national censuses from 2001 and 2010 and the mortality registry from 2001 to 2010. To estimate quality and internal consistency indicators, we used the mortality registry for the entire study period. In Ecuador, by law, the declaration and registration of all deaths is mandatory. The mortality registry contains all death certificates completed in the country by health professionals or (in their absence): 1) police or civil authorities or 2) civil registration officials. Death certificates include sociodemographic information (such as age and sex), home and death addresses, and causes of death. The National Institute of Statistics and Censuses (INEC) uses the information in the certificates to determine the underlying causes of death. For the entire study period, cause of death information was coded using the International statistical classification of diseases and related health problems - 10th Revision (ICD10). All databases were obtained from INEC and are available online (http://www.ecuadorencifras.gob.ec/estadisticas/).

B) Completeness

Completeness can be defined as the percentage of deaths registered in the population covered by the mortality registration system [9]. To estimate completeness, we used a set of demographic methods called death distribution methods (DDMs). DDMs are one of several types of methods to estimate mortality completeness that also include capture-recapture methods, comparison of mortality with other estimates, and a newly developed empirical method that models key drivers of mortality in a population to estimate completeness [11]. DDMs are the most widely used methods to estimate adult mortality completeness. DDMs compare the age distribution of a population at two points (censuses) with the age distribution of the recorded deaths in that population (in the inter–censal period). To estimate mortality completeness, these methods assume *“…that birth and death rates are constant, that there is no net migration in the population, and that the extent of age misreporting and other errors in data collection are minimal*…” [12]. The three main types of DDMS were used: 1) Generalized Growth Balance (GGB); 2) Synthetic Extinct Generations (SEG); and 3) GGB and SEG hybrid (GGBSEG). A complete description of these methods can be found elsewhere [12, 13]. For these estimates, national and provincial population counts and the inter-censal mortality counts for men and women in 18 five-year age groups were obtained from both censuses and the mortality registry. Completeness estimates for male and female mortality in the study areas were estimated using the R package DDM [14]. For each sex and for each method used, DDM automatically chooses the age interval that best fits the models and minimizes the residuals. The age groups used for the estimations can be seen in Table 1. For each study area and for all Ecuador (for both men and women), we present the completeness estimates for each DDM method and the harmonic mean of the three methods. A more detailed description of the methods used can be found in Additional file 1: Appendix 1.

C) Quality

Quality of the mortality registry can be assessed in many ways. Authors have proposed different strategies based on the review of sex and age distribution, of cause of death patterns and the review of ill-defined causes / “garbage codes” [15, 16]. In this study, the quality of mortality registration was assessed by two means: 1) estimating the percentage of “garbage codes” in the registered deaths in each of the study areas; and 2) estimating the percentage of reported deaths with unspecified age or sex.

Garbage codes can be defined as deaths assigned to codes that provide little or no information for cause of death analysis in public health. Naghavi et al. (2010) described four typologies of garbage codes: 1) “Causes that cannot or should not be considered as underlying causes of death”, such as signs and symptoms (R category in the ICD-10) or essential primary hypertension; 2) “Intermediate causes of death”, such as heart failure or peritonitis; 3) “Immediate causes of death that are the final steps in a disease pathway leading to death”, such as cardiac arrest; and 4) “Unspecified causes within a larger cause grouping”, such as diseases where the site is unspecified [17]. We complemented this garbage code list by comparing it with the Global Burden of Disease (GBD) 2013 and 2015 cause lists [18, 19]. This process allowed us to obtain a consistent list of GBD codes and garbage codes. We then estimated the percentage of garbage codes (total and of each of the 4 types) in each of the studied areas for deaths of men and women. The complete list of garbage codes used can be found in Additional file 2: Table S1. Annual percentages of garbage codes were also obtained to describe the temporal evolution in each study area.

Quality indicators were also analysed by age and sex. Age was categorized in the same 20 age groups used in the GBD: early neonatal (0–6 days old), late neonatal (7–27 days old), post-neonatal (28 to 364 days old), 1–4 years; 5-year age groups from 5 to 9 to 75–79 years, and an open age group of 80 years and older [20].

As the quality of death registration can be affected by the professional background of the people registering the deaths [8], the association between the percentage of garbage codes and the percentage of deaths not coded by a medical doctor (MD) was explored visually through the use of scatterplots and quantitatively through the estimation of Pearson’s correlation coefficients and their 95% confidence intervals.

D) Internal consistency

Following previous studies on the subject, the internal consistency of the death registry was assessed by estimating the percentage of deaths with reported causes of death considered impossible in some age–sex combinations [4, 9]. These combinations were created for the study by Phillips et al. (2014) by reviewing medical literature and expert consultation. The list of medically impossible causes of death can be found in Additional file 2: Table S2 [9]. This internal consistency indicator was also estimated for the 20 age groups mentioned previously.

E) Evaluation of the mortality registry in the 22 provinces

Based on the indicators of completeness and garbage coding (quality), we classified the study areas in 4 categories: 1) Acceptable: provinces with completeness of 70% or more, and with 35% or less garbage codes; 2) Deficient in completeness: areas with completeness below 70% but with 35% or less garbage codes; 3) Deficient in quality: areas with completeness of 70% or more, but with more than 35% garbage codes; and 4) Deficient in completeness and quality: provinces with completeness of less than 70%, and with more than 35% garbage codes. The 70% threshold for completeness was used in the study by Mathers et al. (2005) to define medium (> 70%) and low-quality data [6]. For garbage codes, the 35% threshold was used as it is the national percentage for the study period (allowing us to identify study areas above or below the national percentage).

## Results

Of the 762,131 deaths registered in the study period, 330,450 (43.36%) involved women. More than 50% of the deaths registered involved people living in three provinces: Guayas (26.63%), Pichincha (18.13%), and Manabí (9.64%).

A) Completeness

**Fig. 1:**
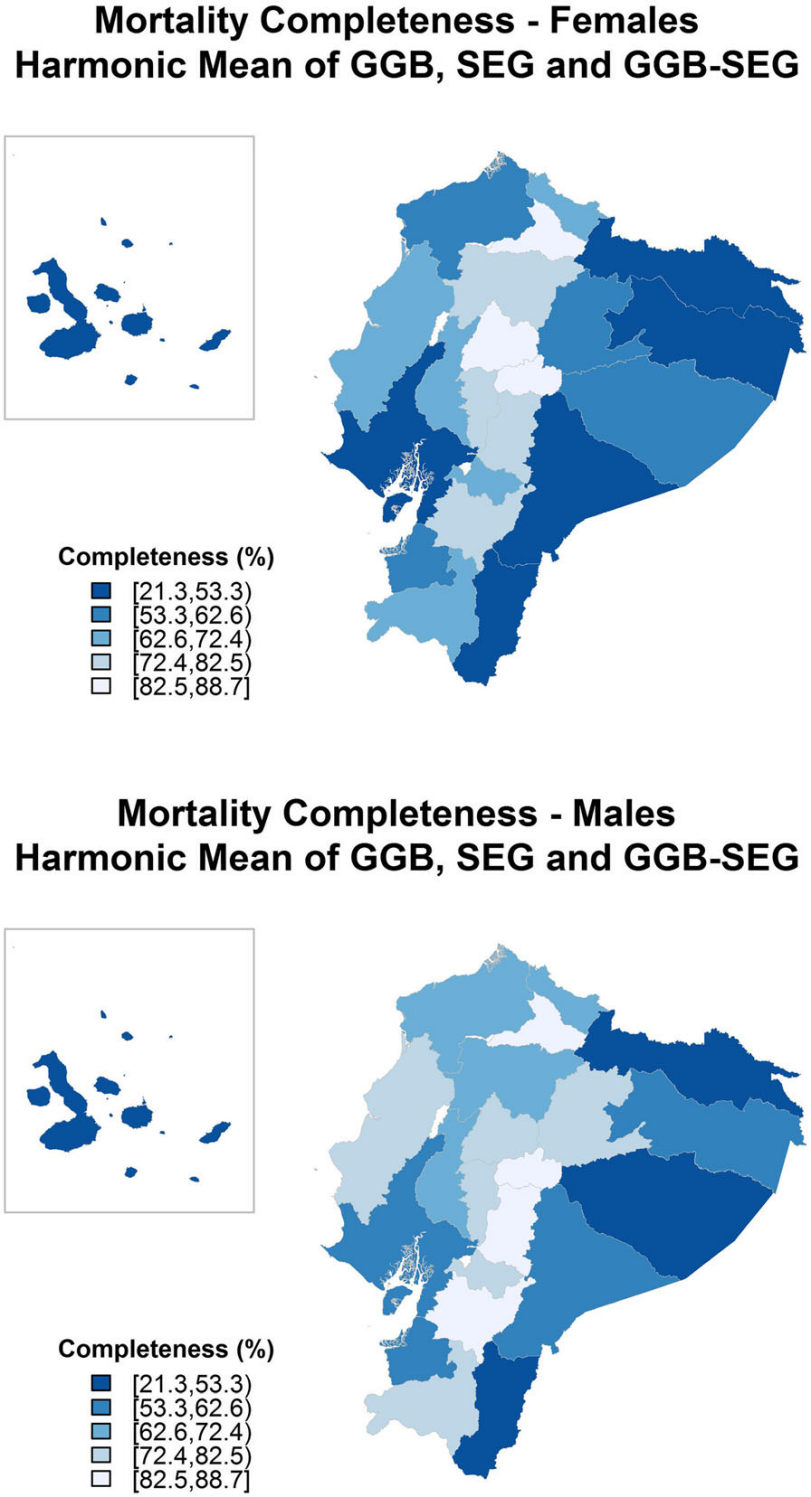
Geographical distribution of mortality data completeness (2001–2010). *GGB*, Generalized growth balance method, *SEG*, Synthetic extinct generations method. GGB-SEG: Hybrid generalized growth balance and synthetic extinct generations method

Differences were found between men and women, as well as wide heterogeneity among areas, in the completeness estimates in Ecuador. Table 1 shows the national and provincial completeness estimates using GGB, SEG, GGBSEG, and the harmonic mean of the three estimates for men and women. At the national level, completeness estimates ranged from 57.2% (GGBSEG) to 71.97% (GGB) in women and from 60.09% (GGBSEG) to 74.68% (SEG) in men. When the 22 provinces were analysed (using the harmonic mean of the three estimators), completeness for women ranged from 21.30% (in Galápagos) to 87.44% (in Tungurahua) and from 34.51% (in Galápagos) to 88.66% (in Chimborazo) for men. Figure 1 shows the geographical pattern of completeness (using the harmonic mean of the three estimates) for men and women. In both sexes, the worst completeness estimates were seen in Galápagos, the Amazonic provinces and in some of the coastal areas.

B) Quality

**Fig. 2:**
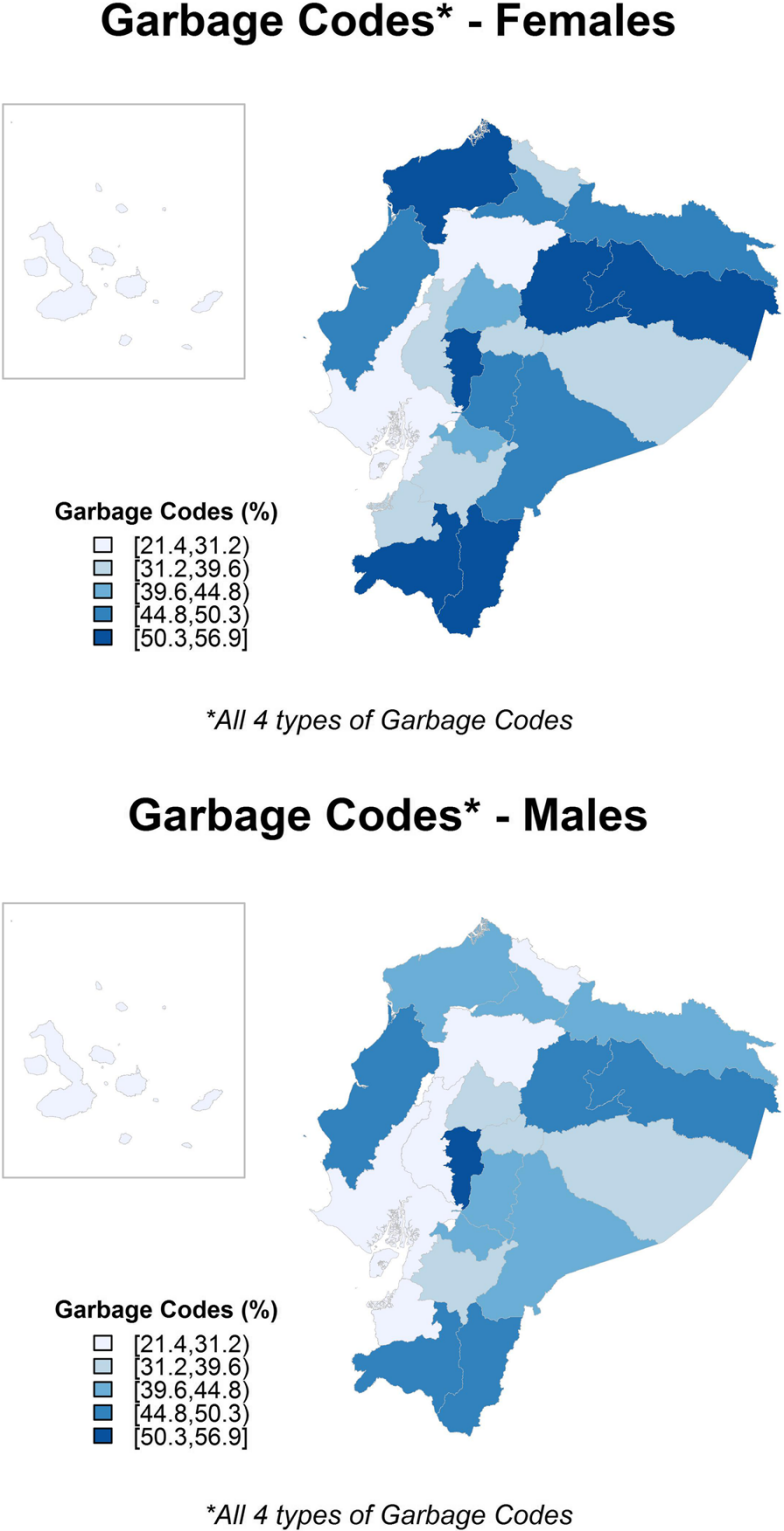
Geographical distribution of garbage codes (2001–2013)

Differences in the quality of the mortality registry were observed between men and women and among the study areas in Ecuador. Table 2 shows the national and provincial garbage code percentages (of each of the 4 types). At the national level, garbage codes were used in 36.67% of the reported deaths in women (15.63% type 1, 11.63% type 2, 1.80% type 3, and 7.61% type 4), and in 34.08% of the reported deaths in men (13.28% type 1, 9.71% type 2, 1.49% type 3, and 9.60% type 4). When the 22 provinces were analysed, the percentage of garbage coding (all 4 types) for women ranged from 21.39% (in Galápagos) to 56.88% (in Bolivar) and from 25.17% (in Galápagos) to 52.05% (in Bolivar) for men. Figure 2 represents the geographical pattern of garbage coding (all 4 types) for men and women. The garbage code percentages were consistently higher for women than for men. In addition, in men and women, the highest garbage code percentages were seen in the Amazonic provinces, in the northern coastal provinces and in the central Andean region.

**Table 2:** Provincial and national garbage code percentages (2001–2013)

Area	Women						Men					
	Deaths 2001–2013	Garbage Codes (%)					Deaths 2001–2013	Garbage Codes (%)				
		Type 1	Type 2	Type 3	Type 4	Total		Type 1	Type 2	Type 3	Type 4	Total
Azuay	18,689	15.43	11.24	0.67	8.16	35.50	21,052	11.90	9.04	0.73	10.14	31.81
Bolivar	6101	35.16	10.95	2.74	8.03	56.88	7250	30.00	9.34	2.48	10.23	52.05
Cañar	6238	24.05	11.46	0.82	7.90	44.23	7426	19.89	9.21	0.81	9.72	39.63
Carchi	4589	5.40	12.42	0.87	12.55	31.24	5322	4.87	8.74	0.79	13.19	27.59
Cotopaxi	12,031	20.50	12.81	1.71	8.49	43.51	14,628	16.11	10.59	1.31	11.14	39.15
Chimborazo	15,579	23.47	14.64	3.74	6.91	48.76	17,401	20.14	12.03	3.03	8.38	43.58
El Oro	12,021	8.93	12.17	2.07	8.47	31.64	17,763	7.23	9.90	1.63	10.35	29.11
Esmeraldas	8823	36.77	6.06	1.25	7.27	51.35	14,189	27.59	4.61	0.92	10.34	43.46
Guayas / Santa Elena	85,727	9.29	12.61	1.72	5.72	29.34	117,259	7.87	11.14	1.54	6.52	27.07
Imbabura	11,952	25.41	8.92	1.20	9.26	44.79	13,773	21.11	7.04	1.01	12.31	41.47
Loja	11,946	32.81	9.72	0.70	7.78	51.01	14,602	30.02	8.99	0.73	9.61	49.35
Los Rios	17,483	12.07	11.97	2.18	7.48	33.70	27,734	9.78	10.11	1.88	8.75	30.52
Manabi	30,544	31.54	9.31	2.99	5.77	49.61	42,916	27.79	7.99	2.39	7.70	45.87
Morona Santiago	2034	28.81	9.73	0.69	6.74	45.97	2740	23.94	9.12	0.44	11.09	44.59
Napo	1600	43.81	5.38	0.38	6.31	55.88	2368	35.68	4.52	0.34	9.71	50.25
Pastaza	1106	14.28	12.03	1.45	8.86	36.62	1622	13.32	12.76	1.48	12.02	39.58
Pichincha / Santo Domingo	62,609	4.23	11.32	1.58	9.91	27.04	75,518	3.33	9.15	1.10	13.91	27.49
Tungurahua	15,522	10.39	17.93	2.11	8.45	38.88	18,011	9.49	14.68	1.63	9.97	35.77
Zamora Chinchipe	1476	41.33	6.84	1.29	5.56	55.02	2081	32.77	6.87	0.48	9.42	49.54
Galapagos	173	9.83	4.62	0.58	6.36	21.39	298	6.38	7.72	3.02	8.05	25.17
Sucumbíos	2018	29.83	5.80	0.99	8.57	45.19	3927	22.94	3.97	0.36	13.57	40.84
Orellana	1651	42.34	3.57	0.61	6.66	53.18	2715	33.37	3.02	0.88	11.90	49.17
Ecuador	*330,450*	*15.63*	*11.63*	*1.80*	*7.61*	*36.67*	*431,681*	*13.28*	*9.71*	*1.49*	*9.60*	*34.08*

Type 1: Causes that cannot or should not be considered as underlying causes of death

Type2: Intermediate causes of death

Type 3: Immediate causes of death that are the final steps in a disease pathway leading to death

Type 4: Unspecified causes within a larger cause grouping

The temporal evolution of garbage coding in all the study areas can be seen in Additional file 3: Figure S1 and Additional file 4: Figure S2. When we analyse the temporal evolution of garbage coding, we can observe that there has been a steady descent in garbage codes in Ecuador in the study period; but that the situation in individual provinces is heterogeneous. Provinces located in the Amazonic, northern Coastal and in the central Andean regions show consistently high garbage code percentages through the study period. Other provinces show important declines in garbage code percentages. For example, provinces in the southern Coastal region (Guayas/Santa Elena, Los Rios and El Oro) and Azuay in the Andean region show this trend.

The age pattern of deaths coded with garbage codes showed a clear age gradient in both men and women: with percentages being lower at younger ages (7.01% in the early neonatal age group) and higher in the older age groups (42.64% in the group of people aged 80 years or older). The age pattern of garbage codes can be seen in detail in Additional file 2: Table S3.

The percentage of deaths not registered by an MD ranged from 1.5% (in Pichincha/Santo Domingo) to 50.17% (in Napo) for men; and from 1.03% (in Pichincha/Santo Domingo) to 53.12% (in Napo) for women. In all areas and in both men and women the percentages of garbage codes were higher than the percentage of deaths not coded by an MD. Additional file 5: Figure S3 shows visually how the percentage of deaths not coded by an MD relate to the percentage of garbage codes in the study areas. It can be noticed that many provinces in the Amazonic region and one province in the northern Coastal region (Esmeraldas) have high percentages of deaths not coded by an MD and high percentages of garbage codes. Also, some provinces in the Andean region (and Manabí in the coastal region) have high percentages of garbage codes, even thou their percentages of deaths not coded by an MD is lower. Pearson’s correlation was 0.82 (95%CI 0.61 to 0.92) for men and 0.84 (95%CI 0.65 to 0.93) for women.

When we analysed deaths without age or sex information, we found that all the registered deaths in the study period had sex information and that 948 (0.12%) had no age information (608 men and 240 women). More than half of the deaths with no age information were registered in three provinces: Pichincha (288 deaths), Azuay (124 deaths) and Loja (118 deaths).

C) Internal consistency

At the national level, only 0.23% of the registered deaths in the mortality registry had causes of death considered impossible for the age or sex of the deceased. These percentages were similar in men (0.23%) and women (0.24%). In all but one of the areas, the percentage of deaths with causes of death considered impossible for the age / sex combinations was less than 1% (the exception was Galápagos were the percentage for men was 1.01%). The age pattern of these reported deaths showed that, for both men and women, their percentage was much higher at younger ages; especially in deaths occurring in children younger than 1 year (5.63% of the registered deaths in the age group between 28 days and 1 year). The age pattern of deaths with a reported cause impossible for the age/sex of the deceased is shown in detail in Additional file 2: Table S4.

D) Mortality registry assessment at the provincial level

**Fig. 3:**
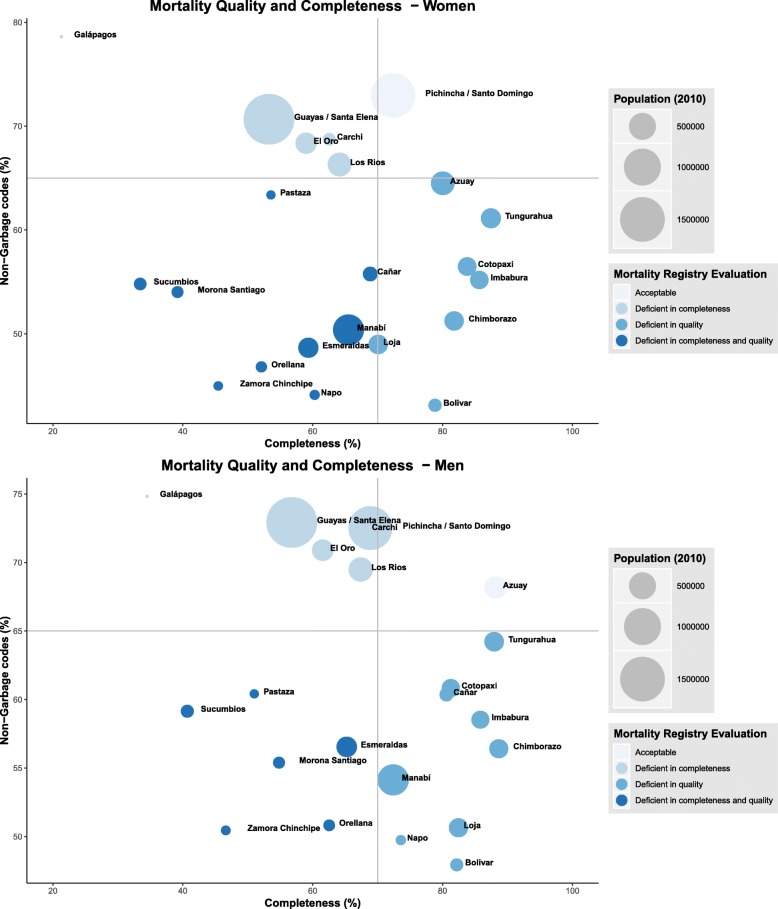
Evaluation of the mortality registry at the provincial level .Completeness: Harmonic mean of the three methods used for completeness estimations

Figure 3 shows the classification of each province considering the mortality registry completeness and a quality indicator (percentage of non-garbage codes) for men and women. The size of each dot in the figure represents the population (in the 2010 census). In both men and women, only one area was classified as acceptable (Pichincha/Santo Domingo for women and Azuay for men). Of note, for both men and women, most of the areas located in the coastal region (except Manabí and Esmeraldas) and Galápagos were deficient in completeness; most of the areas located in the Andean region were classified as deficient in quality and almost all of the areas located in the Amazonic region were considered deficient in completeness and quality.

## Conclusions

We evaluated the completeness, quality and internal consistency of the mortality registry in the provinces of Ecuador from 2001 to 2013. Our results show that there are substantial inequalities in the registry by sex, geographical area and age. Completeness was lower for women in 20 of the 22 studied areas. The same was true for the country as a whole. The geographical pattern of completeness – for both men and women – showed that Galápagos and the Amazonic provinces had lower completeness estimates. Analysis of the quality of the mortality registry showed that the percentage of garbage codes was higher for women in 19 of the 22 study areas and in all of Ecuador. Analysis of the types of garbage codes showed that the percentages of types 1 to 3 were consistently higher in women and type 4 was more frequent in men. The geographical pattern of garbage coding showed that these codes were more common in the Amazonic provinces, in the central Andean region and in the northern coastal region. These areas also showed little or no improvement in garbage code percentages during the study period. In both sexes, there was a positive correlation between the percentage of deaths not coded by an MD and the percentage of garbage codes. For both sexes, there was also a clear age gradient in garbage coding, the percentage being much higher at older age groups. Internal consistency was high in all the studied areas. Nevertheless, we found that there was also an age gradient for internal consistency, which was lower in younger age groups. Classification of the areas by the completeness and quality of the mortality registry revealed that the mortality registry could not be classified as acceptable for both sexes in any of the study areas (Pichincha/Santo Domingo was acceptable for men and Azuay for women).

The literature reviewed shows wide heterogeneity between the mortality registry completeness estimates reported for Ecuador. This can be attributed to the diversity of the methods that can be used to estimate completeness; methods that are rarely made explicit in the reports from many international health organizations. Murray et.al compared 234 variants of DDMs and recommends using combinations of GGB, SEG and GGBSEG for estimations, a recommendation we followed [12]. Our results are in agreement with those reported by Phillips et al. (Additional file 3: Figure S1), in which the completeness of the mortality registry in Ecuador seems to have a decreasing tendency and in 2010 was between 60 and 70% [9]. Nevertheless, our results are different from the ones in other studies. For example, the UN population division estimates completeness to be a little higher (between 70 and 79%), and the GBD 2016 project estimates a mortality completeness over 80% for the study period [21, 22]. The differences found between the different sources can be explained by the use of diverse methods to estimate completeness and the high uncertainty of the methods. For example, the GGB project uses the three DDM methods as recommended by Murray et al. (2010) to estimate adult mortality. After that, they combine the results from the DDM methods with estimations of under-5 completeness for several time periods. When necessary, they use sibling histories too. Even after all this complex process, they still recognize that estimates may change between GBD revisions [23].

To further compare our results to the ones obtained using different methods, we obtained national and provincial completeness estimates for 2010, applying the empirical method described by Adair and López (2018). This method uses crude death rates, the population age structure, and the under-five mortality rate and registration completeness as predictors of random-effects models that estimate mortality completeness [11]. The method makes two fairly strong assumptions, namely that deaths occur in the sub-national areas of residence and that the estimates of populations are accurate. Moreover, authors state that the models used could perform poorly in countries experiencing high HIV prevalence or high number of violent or alcohol related deaths. This method estimates (for all Ecuador) a completeness over 90% for both sexes; a result that seems unusually high if compared to the rest of estimates of the country (ranging from 60 to 80%).

Moreover, when we estimated the provincial results (see Additional file 2: Table S5), we could observe that some of the most rural provinces (in the Amazonic region), with lowest data quality, and less deaths certified by a medical doctor have high completeness estimates. For example, Amazonic provinces like Napo, Zamora Chinchipe and Sucumbíos have higher completeness estimates than Pichincha (province with mostly urban population and much more accessibility to health services). These results are in concordance to what was found when obtaining sub-national completeness estimates in Brazilian states, where concordance was lower when comparing GGB and the new method. These abnormal results could be explained by: 1) unreliable under-five mortality estimates; 2) unreliable population estimates; 3) poor performance of the method in subnational settings, especially when data quality is low. Certainly, much work is still needed to improve completeness estimation methods worldwide, especially in sub-national contexts.

As mentioned previously, geographical inequalities in the completeness and quality of mortality registries have been seen in other countries in South America. In our study, the pattern of completeness and garbage coding is similar to the pattern of socio-economic deprivation in Ecuador [24]. Moreover, some of the provinces with worst completeness and quality indicators are those with the highest proportions of ethnic groups that have historically been excluded and impoverished (indigenous, afro-ecuadorian, and montubio) [25]. When analysing the temporal evolution of the quality of the mortality registry, these areas show little or no improvements. Attention should be brought also to the provinces that paradoxically have high quality and low completeness or high completeness and low quality of the mortality data. Galapagos has a low percentage of garbage codes (high quality) and also a low and also a low completeness estimate. We believe that this could be explained by the low population (and consequent low number of deaths) and relatively high migration rates in the Province that could make DDM methods to perform poorly (completeness being underestimated). On the other side, many provinces in the central Andean region show high mortality completeness estimates and low-quality data. We believe that the particular characteristics of these areas should be studied more in depth in order to explain this phenomenon.

Inequalities in the mortality registry quality and completeness between men and women have scarcely been studied in low- and middle-income countries. In Brazil, differences in completeness and ill-defined causes have been found between men and women [26]. There could be some explanations for the differences found in this study: 1) Gender bias in the diagnosis of various conditions has been reported for many conditions in other countries [27, 28], which could also be an important issue in Ecuador. 2) Because more men die from external causes (which require investigation of the death and autopsy), their deaths could be registered more frequently and with more correct diagnoses. This has been proposed as an explanation for the sex differences in the mortality registry quality observed in Belo Horizonte, Brazil [29]. 3) In other contexts, maternal deaths have been found to be greatly underreported and misclassified [30, 31]. All of these explanations should be studied in greater depth in Ecuador.

The correlation between the professional background of the people who certify deaths and the quality of the mortality registry has been observed in other countries in the region; finding that mostly rural areas have less deaths certified by medical doctors and lower quality of the mortality data [8]. We have found a similar pattern in Ecuador, especially in the provinces in the Amazonic region and Esmeraldas in the Coastal region. Populations in provinces with disperse populations in rural areas could have greater difficulties finding healthcare personnel to certify deaths. One thing that can be noticed is that there is still an important percentage of deaths certified by medical doctors and assigned to garbage codes, especially in many provinces in the Andean region. This means that work is needed not only to improve access to healthcare professionals, but also to improve the quality of death certification by medical doctors.

The age pattern of garbage codes was similar to that observed in other contexts, and could be explained by the difficulty of determining the cause of death accurately in older people (with multiple comorbidities) [32]. For example, we analysed the principal garbage codes in people over 80 years old and found that heart failure (ICD10 code I50), senility (ICD10 code R54) and essential hypertension (ICD10 code I10) accounted for approximately 40% of the garbage codes in that age group. Deaths coded as heart failure accounted for 17.21% of the garbage codes in women and 16.30% in men; senility accounted for 15.34% of garbage codes in women and 13.55% in men; and essential (primary) hypertension accounted for 10.54% of garbage codes in women and 9.76% in men. Even though the internal consistency indicator was low in all of the studied areas, we believe it is important to also notice the age pattern. More research is needed to assess why more “medically impossible” causes of death are assigned at younger ages in Ecuador.

Our study has some limitations. The most important ones relate to the limitations of the estimation of completeness by DDMs. These methods assume that: 1) the studied population is closed to migration; 2) that completeness (of mortality and census counts) are constant in all age groups; and 3) that age misreporting is minimal or non-existent [12]. All these issues could be important, but migratory patterns could be a vital factor in all middle- and low-income countries and especially in Ecuador during the study period (with substantial internal and external migratory waves) [33, 34]. Royuela & Ordoñez (2018) describe the internal migration patterns in the Provinces of Ecuador, using data from the national censuses since 1982. They describe important differences between provinces. For example, in 2010, percentages of net migration ranged from − 8.7 (Bolivar) to 10.9 (Galápagos). We believe that some of the completeness estimates could be underestimated (especially in Guayas and Pichincha) due to this problem. Some authors have proposed approaches to adjust some of the DDM methods for migration [35], but their use is still not widespread. In the future, methods that include migration could be used in the Ecuadorian context. Other important limitation of DDM methods is uncertainty. Murray et al. (2010) reported that “…the uncertainty around relative completeness of registration is likely to be at least +/-20% of the estimated level, and perhaps considerably more…” [12]. In Additional file 1: Appendix 1, we compare completeness estimates presented in the article with estimates obtained using the age groups recommended in the article by Murray and colleagues. We found that, at a national level, results were quite similar between the two approaches (3.43% difference between harmonic mean of the three methods in women and 5.51% in men). Nevertheless, results showed a much greater variability in the provincial estimates. All this means that the completeness estimates presented should be taken as rough estimates, helpful to orientate public policy and have a sense of the geographical pattern of completeness in the country; but not as definitive and precise estimates. Greater work is needed to refine methods to estimate completeness at subnational levels and in countries were data is not completely compliant with the assumptions of DDM methods. As Hill (2017) points out, even thou in the last decade important developments in analytical strategy have been seen, “…very little in the way of new methodology has emerged…” [13]. New methods are scarce, but they have the potential of dealing with some of the limitations of DDMs as the lack of timeliness, complexity and the assumption of no migration. Certainly, there still much work needed in order to have reliable subnational completeness estimates for many middle and low income countries. Another important step to obtain better estimates of mortality completeness by age groups would be to use indirect methods to assess completeness in other age groups (such as infants). Nevertheless, our results provide a good idea of adult mortality completeness and can be used to adjust adult mortality estimates in the future. Regarding the quality and internal consistency indicators, we believe it could be important to assess and adapt the garbage code and “medically impossible” cause lists to the local context. We should also consider that garbage codes, age/sex missingness and internal consistency are proxy indicators of the overall quality of the mortality registry. Other important quality issues as classification accuracy could not be measured in this study.

Our study is the first comprehensive evaluation of the mortality registry in Ecuador and of the inequalities (geographical, age, sex) it contains. The results of this study will be important for public health researchers, planners of national statistics and for the general population. In particular, Ecuador’s National Institute for Statistics and Censuses (INEC) can use this research to determine which areas and populations require greater attention to strengthen and improve the registry and whether it should focus on completeness, quality, or both. If we consider vital statistics systems as “essential public goods” [1], which help us to better understand our societies and tackle inequalities and other important problems, it is imperative to invest in their continuous improvement and maintenance.

### Concluding remark

We found that the mortality registry in Ecuador has a series of limitations, which were more substantial in some areas than others. Nonetheless, we believe that the efforts and resources invested in assembling and maintaining the registry should not be lost. We also believe that mortality data in Ecuador can be used to study health inequalities but that, to do so, some additional work is required. Adjustment of mortality data by completeness and the use of redistribution protocols for garbage codes have already been effectively employed in the GBD study [22]. A similar approach can be adopted at a sub-national level to correct mortality estimates and cause of death data until the vital statistics system achieves an optimal level of completeness and quality in the registration of deaths.

## Additional files

Additional file 1 : **Appendix 1.** Death Distribution Methods (DDM) Used in the Study (PDF 247 kb)

Additional file 2 : **Table S1.** List of ICD-10 codes considered garbage codes by type**. Table S2.** Causes of death considered impossible in some age – sex combinations**. Table S3.** Distribution of garbage code percentages (2001–2013) by age groups**. Table S4.** Distribution of causes of death considered impossible in some age – sex combinations (2001–2013) by age groups**. Table S5.** Completeness estimates using Adair and López (2018) method - 2010 (XLSX 27 kb)

Additional file 3 : **Figure S1.** Evolution of garbage code percentages (2001–2013) in each of the study areas - Women (PDF 10 kb)

Additional file 4 : **Figure S2.** Evolution of garbage code percentages (2001–2013) in each of the study areas – Men (PDF 10 kb)

Additional file 5 : **Figure S3.** Garbage Code percentages and percentages of deaths not coded by a Medical Doctor (2001–2013) for Women and Men – Scatterplot (PDF 6 kb)
