# Correction to: Evaluation of the mortality registry in Ecuador (2001–2013) – social and geographical inequalities in completeness and quality

**DOI:** 10.1186/s12963-019-0185-9

**Published:** 2019-04-18

**Authors:** Andrés Peralta, Joan Benach, Carme Borrell, Verónica Espinel-Flores, Lucinda Cash-Gibson, Bernardo L. Queiroz, Marc Marí-Dell’Olmo

**Affiliations:** 1 Agència de Salut Pública de Barcelona, Plaça Lesseps 1, 08023 Barcelona, Catalonia Spain; 2 Health Inequalities Research Group, Employment Conditions Knowledge Network (GREDS-EMCONET), Department of Political and Social Sciences, Universitat Pompeu Fabra, Ramon Trias Fargas, 25-27, 08005 Barcelona, Spain; 3 Johns Hopkins University, Pompeu Fabra University Public Policy Center, Barcelona, Spain; 4 Transdisciplinary Research Group on Socioecological Transitions (GinTRANS2), Universidad Autónoma Madrid, 28049 Madrid, Spain; 5 CIBER Epidemiología y Salud Pública (CIBERESP), Madrid, Spain; 6 Institut d’Investigació Biomèdica (IIB Sant Pau), Barcelona, Spain; 7 Department of Experimental and Health Sciences, Universitat Pompeu Fabra, Barcelona, Catalonia Spain; 8 Department of Demography and Cedeplar, Faculdade de Ciências Econômicas, FACE/UFMG, Campus Pampulha, Av. Antônio Carlos, 6627, Pampulha, Belo Horizonte, MG CEP 31270-901 Brazil

**Correction to: Popul Health Metrics (2019) 17:3**

**https://doi.org/10.1186/s12963-019-0183-y**

Following the publication of this article [1], the authors reported a typesetting error in Table 1 that caused the columns of the table to be ordered incorrectly, and a typographical error in a sentence in the Conclusions section.

The incorrect Table 1 was published as:

**Table 1:** Provincial and national completeness estimates (2001 – 2010)

Area	Women											Men				
	Population 2001	Population 2010	Deaths IC Period	Age Range Used	Population 2001	Population 2010	Deaths IC Period	GGB^a^	Age Range Used	SEG^b^	GGB - SEG^c^	Mean	GGB ^a^	SEG ^b^	GGB - SEG ^c^	Mean
Azuay	319,974	375,027	12,730	98.82	80.50	66.91	80.03	15–50	279,339	335,739	14,465	107.10	91.49	72.69	88.17	15–50
Bolivar	86,727	93,767	4280	98.81	71.91	71.29	78.83	20–60	83,969	89,975	5100	95.56	79.52	74.28	82.18	25–60
Cañar	111,707	119,621	4187	99.87	57.49	61.89	68.86	20–55	94,639	104,812	5137	95.54	79.06	70.87	80.59	15–50
Carchi	76,891	83,257	3147	80.20	56.76	55.98	62.56	15–50	75,106	80,905	3800	85.18	60.80	62.58	67.92	15–50
Cotopaxi	180,050	209,723	8369	99.92	83.67	72.23	83.79	20–55	169,507	197,990	10,264	101.66	84.40	65.66	81.27	15–50
Chimborazo	213,297	239,339	10,896	96.01	78.20	74.17	81.78	20–55	191,150	219,221	12,414	95.61	93.55	78.81	88.66	20–55
El Oro	258,108	295,256	8020	76.86	55.03	50.83	58.99	15–50	265,561	302,735	12,037	68.33	62.60	55.16	61.55	15–50
Esmeraldas	188,213	262,143	5758	43.07	86.01	63.62	59.33	15–50	197,331	270,912	9675	43.94	106.46	72.29	65.24	15–50
Guayas / Santa Elena	1,654,813	1,975,927	57,031	57.04	57.70	46.62	53.27	15–50	1,643,493	1,968,672	79,288	54.37	67.21	51.12	56.79	15–50
Imbabura	176,064	204,094	8243	101.18	86.67	73.53	85.66	15–55	167,689	193,105	9597	100.32	86.91	74.17	85.82	20–60
Loja	207,913	228,704	8141	100.81	62.41	59.31	70.09	15–50	198,316	221,638	9962	101.55	79.32	71.79	82.45	15–50
Los Rios	315,609	379,883	12,055	75.27	68.77	52.78	64.14	15–50	335,909	398,252	19,140	70.13	77.06	57.89	67.40	15–50
Manabi	592,524	681,575	20,872	82.71	63.62	55.61	65.51	15–50	599,521	689,525	29,639	85.19	74.86	61.20	72.40	15–50
Morona Santiago	57,891	73,126	1327	46.48	39.80	33.43	39.19	15–50	56,904	74,529	1806	54.03	63.17	49.05	54.82	15–50
Napo	38,776	50,641	1029	62.22	67.23	53.19	60.31	15–50	39,965	52,220	1577	71.98	88.74	64.05	73.57	15–50
Pastaza	29,686	41,394	718	40.59	73.71	56.18	53.57	15–50	31,808	42,084	1053	45.81	61.87	48.02	51.01	15–50
Pichincha / Santo Domingo	1,218,916	1,504,023	42,824	72.07	81.98	65.03	72.37	20–55	1,167,281	1,441,529	52,093	64.46	81.18	63.62	68.89	15–50
Tungurahua	227,308	258,907	10,747	98.69	83.24	82.22	87.44	15–55	213,470	244,014	12,569	99.48	87.86	78.87	87.94	15–55
Zamora Chinchipe	37,017	43,780	976	53.53	45.88	39.20	45.46	15–50	39,462	46,627	1391	47.16	52.06	41.78	46.62	15–50
Galapagos	8137	11,392	123	12.58	35.10	30.43	21.30	15–50	9618	12,238	187	25.46	45.66	38.83	34.51	15–50
Sucumbíos	58,774	83,431	1279	22.00	49.52	41.57	33.45	15–50	67,901	91,050	2589	28.53	58.70	46.24	40.70	15–50
Orellana	39,478	64,034	1073	28.85	105.65	74.30	52.10	15–50	45,147	70,655	1766	36.30	126.62	79.87	62.54	15–50
Ecuador	6,132,183	7,293,907	224,300	71.97	67.67	57.21	65.00	15–50	6,010,246	7,165,170	296,474	71.76	74.68	60.09	68.23	15–50

^a^ Generalized growth balance method

^b^ Synthetic extinct generations method

^c^ Hybrid generalized growth balance and synthetic extinct generations method

The correct Table 1 should be:

**Table 1:** Provincial and national completeness estimates (2001 – 2010)

Area	Women								Men							
	Population 2001	Population 2010	Deaths IC Period	Completeness (%)				Age Range Used	Population 2001	Population 2010	Deaths IC Period	Completeness (%)				Age Range Used
				GGB^a^	SEG^b^	GGB - SEG^c^	Mean					GGB^a^	SEG^b^	GGB - SEG^c^	Mean	
Azuay	319,974	375,027	12,730	98.82	80.50	66.91	80.03	15 - 50	279,339	335,739	14,465	107.10	91.49	72.69	88.17	15 - 50
Bolivar	86,727	93,767	4,280	98.81	71.91	71.29	78.83	20 - 60	83,969	89,975	5,100	95.56	79.52	74.28	82.18	25 - 60
Cañar	111,707	119,621	4,187	99.87	57.49	61.89	68.86	20 - 55	94,639	104,812	5,137	95.54	79.06	70.87	80.59	15 - 50
Carchi	76,891	83,257	3,147	80.20	56.76	55.98	62.56	15 - 50	75,106	80,905	3,800	85.18	60.80	62.58	67.92	15 - 50
Cotopaxi	180,050	209,723	8,369	99.92	83.67	72.23	83.79	20 - 55	169,507	197,990	10,264	101.66	84.40	65.66	81.27	15 - 50
Chimborazo	213,297	239,339	10,896	96.01	78.20	74.17	81.78	20 - 55	191,150	219,221	12,414	95.61	93.55	78.81	88.66	20 - 55
El Oro	258,108	295,256	8,020	76.86	55.03	50.83	58.99	15 - 50	265,561	302,735	12,037	68.33	62.60	55.16	61.55	15 - 50
Esmeraldas	188,213	262,143	5,758	43.07	86.01	63.62	59.33	15 - 50	197,331	270,912	9,675	43.94	106.46	72.29	65.24	15 - 50
Guayas / Santa Elena	1,654,813	1,975,927	57,031	57.04	57.70	46.62	53.27	15 - 50	1,643,493	1,968,672	79,288	54.37	67.21	51.12	56.79	15 - 50
Imbabura	176,064	204,094	8,243	101.18	86.67	73.53	85.66	15 - 55	167,689	193,105	9,597	100.32	86.91	74.17	85.82	20 - 60
Loja	207,913	228,704	8,141	100.81	62.41	59.31	70.09	15 - 50	198,316	221,638	9,962	101.55	79.32	71.79	82.45	15 - 50
Los Rios	315,609	379,883	12,055	75.27	68.77	52.78	64.14	15 - 50	335,909	398,252	19,140	70.13	77.06	57.89	67.40	15 - 50
Manabi	592,524	681,575	20,872	82.71	63.62	55.61	65.51	15 - 50	599,521	689,525	29,639	85.19	74.86	61.20	72.40	15 - 50
Morona Santiago	57,891	73,126	1,327	46.48	39.80	33.43	39.19	15 - 50	56,904	74,529	1,806	54.03	63.17	49.05	54.82	15 - 50
Napo	38,776	50,641	1,029	62.22	67.23	53.19	60.31	15 - 50	39,965	52,220	1,577	71.98	88.74	64.05	73.57	15 - 50
Pastaza	29,686	41,394	718	40.59	73.71	56.18	53.57	15 - 50	31,808	42,084	1,053	45.81	61.87	48.02	51.01	15 - 50
Pichincha / Santo Domingo	1,218,916	1,504,023	42,824	72.07	81.98	65.03	72.37	20 - 55	1,167,281	1,441,529	52,093	64.46	81.18	63.62	68.89	15 - 50
Tungurahua	227,308	258,907	10,747	98.69	83.24	82.22	87.44	15 - 55	213,470	244,014	12,569	99.48	87.86	78.87	87.94	15 - 55
Zamora Chinchipe	37,017	43,780	976	53.53	45.88	39.20	45.46	15 - 50	39,462	46,627	1,391	47.16	52.06	41.78	46.62	15 - 50
Galapagos	8,137	11,392	123	12.58	35.10	30.43	21.30	15 - 50	9,618	12,238	187	25.46	45.66	38.83	34.51	15 - 50
Sucumbíos	58,774	83,431	1,279	22.00	49.52	41.57	33.45	15 - 50	67,901	91,050	2,589	28.53	58.70	46.24	40.70	15 - 50
Orellana	39,478	64,034	1,073	28.85	105.65	74.30	52.10	15 - 50	45,147	70,655	1,766	36.30	126.62	79.87	62.54	15 - 50
*Ecuador*	*6,132,183*	*7,293,907*	*224,300*	*71.97*	*67.67*	*57.21*	*65.00*	*15 - 50*	*6,010,246*	*7,165,170*	*296,474*	*71.76*	*74.68*	*60.09*	*68.23*	*15 - 50*

^a^Generalized growth balance method

^b^Synthetic extinct generations method

^c^Hybrid generalized growth balance and synthetic extinct generations method

In the Conclusion section the incorrect sentence was:

“Galapagos has a low percentage of garbage codes (high quality) and also a low and also a low completeness estimate.”

It should say:

“Galapagos has a low percentage of garbage codes (high quality) and also a low completeness estimate.”

The original article has been corrected. The publisher apologizes to the readers and the authors for any inconvenience caused by these errors.
