# Supplementary material for: Evaluation of the mortality registry in Ecuador (2001–2013) – social and geographical inequalities in completeness and quality
Source: Popul Health Metr. 2019 Mar 28;17:3. doi: 10.1186/s12963-019-0183-y (PMC6437878; doi:10.1186/s12963-019-0183-y)
Supplement: Supplementary file 1 — : Appendix 1. Death Distribution Methods (DDM) Used in the Study (PDF 247 kb) [file 12963_2019_183_MOESM1_ESM.pdf]

Supplementary Appendix 1 – Death Distribution Methods (DDM) Used in the Study

1. ¿What are DDM Methods?

Death Distribution Methods are a set of demographical methods used for the estimation of the completeness of mortality (and census) data. The most widely used DDMs estimate the average completeness of death registration in the period between two censuses. These methods compare the age distribution of a population at two points (censuses) with the age distribution of the recorded deaths in that population (in the inter–censal period). These DDM methods require age and sex distribution of the population at two points in time (censuses) and the average annual deaths (or total deaths) during the intercensal period (1).

2. Types of DDMS

Three types (or families) of DDM methods are the most widely used for mortality completeness estimations: 1) Generalized Growth Balance (GGB); 2) Synthetic Extinct Generations (SEG); and 3) a GGB and SEG hybrid (GGB-SEG). The following table, adapted from Murray et al. (2010) summarizes the basic concepts in each method (2):

<table>
<tr><td>GGB</td><td>Hill, 1987</td></tr>
<tr><td colspan="2">Base Concept: The mathematical relationship of the demographical balancing equation:

Birth Rate = growth rate + death rate

The slope and intercept of the modeled equation (plotting birth rate versus death rate) along with observed growth rates can be used to obtain the relative coverage of census 1 to census 2, as well as the relative completeness of death registration to census coverage.</td></tr>
<tr><td>SEG</td><td>Bennett and Horiuchi, 1981 and 1984</td></tr>
<tr><td colspan="2">Base Concept: The number of people at age x at time 0 is equal to the number of deaths age x in year 0, plus deaths age x+1 in year 1, plus deaths age x+2 in year 2 and so on until the entire cohort is extinct. SEG uses intercensal age-specific growth rates and current deaths at older ages to estimate future cohort deaths.

By comparing estimated future cohort deaths to current cohort size, the completeness of death registration can be estimated.</td></tr>
<tr><td>GGB-SEG (Hybrid)</td><td>Hill and Choi, 2004</td></tr>
<tr><td colspan="2">Base Concept: Use GGB to estimate coverage of census 2 relative to census 1 and use this to adjust populations prior to use in SEG method.</td></tr>
</table>

Adapted from Murray et al. (2010).

Further information on the specifics of DDM types can be found in the supplementary text on the Murray et al. paper cited above or in the summary made for the UN Population Division by Hill in 2017 (1). There are other indirect methods that assume a stable population - as the Brass Growth Balance Method and the Preston-Coale method - but they are not widely used as their assumptions (stable populations) are source of important bias (3). The description of these methods is beyond the scope of this appendix.

3. Assumptions of DDMs

DDMs make the following assumptions: 1) that the population is closed to migration; 2) that the completeness of recording of deaths is constant by age; and 3) that the completeness of population recording is constant by age, and that ages of the living and the dead are reported without error (4). Moreover, SEG makes the assumption of constant coverage across the two censuses; and GGB-SEG assumes that the relative coverage of censuses is constant by age (2).

DDM methods usually perform well when the data used for estimations is compliant when their assumptions. The three methods are especially sensitive to migration. Adjustment of the methods for migration has been proposed, but its limited because: 1) migration statistics are scarce or incomplete in many countries; and 2) the adaptation of the methods when migration is not known (4) are still not completely developed and accepted (1).

4. Strategies to minimize bias IN DDMs

To try to minimize bias induced by non-compliance with the assumptions of DDMs, researchers have adopted various strategies, many times subjectively. One of the main strategies is to use an age range that is more compliant with the assumptions of the methods. To minimize the effect of migration, some authors have preferred to use age ranges considered to be affected less by migration (over 60 years old for example). Other authors have used simulated data or comparisons of methods to real data in places in which complete registration is presumed in order to determine the appropriate age ranges to be used (1,2,5). Hill, You and Choi (2009) conclude that the best strategy is to use GGB-SEG with an age range of 5 to 65 years old. On the other hand, Murray et al. (2010) conclude the most appropriate strategy is to combine the three types of DDMs using the following age trims: 55 to 80 years old for SEG, 40 to 70 years old for GGB, and 50 to 70 years old for GGBSEG. Other approaches to select the appropriate age trims are based on graphical analyses for each DDM method. The DDM R package presents a solution that automates the choice of age groups for users (6). The main idea is to provide an estimator that will give the best solution from all possible combinations of age ranges. The estimator implemented by the DDM R package is the Root Mean Square Error (RMSE). The Root Mean Square Error, also called the root mean square deviation, is a measure of the difference between values predicted by a model and the values actually observed. These individual differences are also called residuals, and the RMSE serves to aggregate them into a single measure of predictive power. For each individual area, the age range that minimizes the RMSE is taken as the age range for the GGB coverage estimate, and this criterion is designed to mimic the more traditional eyeball fitting used for the GGB method. In other words, the automatic solution is to determine death registration coverage based on the age range that produces the smallest residual difference between observed and estimated death rates and their adjustment line (6).

5. Limitations of the DDM methods

Apart from the potential biases that could happen when data is not compliant with the assumptions of the methods; DDMs have certain limitations that are worth noticing:

1) **Lack of timeliness**: DDM methods depend on information from national censuses and give completeness estimates for the whole intercensal period. This makes completeness estimates inadequate to study rapid or medium term evolutions in death registry completeness. The next census in Ecuador is planned for 2020. This means that only by then we can get a sense of the completeness of death registration in the current decade.
2) **Uncertainty**: Murray et al. (2010) reported that "…the uncertainty around relative completeness of registration is likely to be at least +/-20% of the estimated level, and perhaps considerably more." This means that the completeness estimates presented should be taken as rough estimates, helpful to orientate public policy and have a sense of the geographical pattern of completeness in the country; but not as definitive and precise estimates.

Regardless of all their limitations, DDMs are still some of the most widely used methods for estimating mortality completeness.

6. Sources of Information

To estimate completeness, we used Ecuador's national censuses from 2001 and 2010 and the mortality registry from 2001 to 2010. The 2001 census was performed in November 25, 2001; 12.156.608 people were included in the censal data. The 2010 census was performed in November 28, 2010; 14.483.499 people were included in the censal data (7). In Ecuador, by law, the declaration and registration of all deaths is mandatory. The mortality registry contains all death certificates completed in the country by health professionals or (in their absence): 1) police or civil authorities or 2) civil registration officials. Death certificates include sociodemographic information (such as age and sex), home and death addresses, and causes of death. The National Institute of Statistics and Censuses (INEC) uses the information in the certificates to determine the underlying causes of death. For the entire study period, cause of death information was coded using the International statistical classification of diseases and related health problems - 10th Revision (ICD10). All databases were obtained from INEC and are available online (http://www.ecuadorencifras.gob.ec/estadisticas/).

National and provincial population counts and the inter-censal mortality counts for men and women in 18 five-year age groups were obtained from both censuses and the mortality registry.

7. Analysis

The R DDM package, developed by Tim Riffe, Everton Lima and Bernardo Queiroz was used to estimate completeness using the 3 DDM methods. The R DDM package manual describes the methods used as follows (6):

GGB:

"The method is based on finding a best-fitting linear relationship between two modeled parameters (right term and left term), but the fit, and resulting coverage estimate, depend on

exactly which age range is taken. This function either finds a nice age range for you automatically, or you can specify an exact vector of ages."

SEG:

"The method estimates age-specific degrees of coverage. The age pattern of these is assumed to be noisy, so we take the arithmetic mean over some range of ages. One may either specify a particular age-range, or let the age range be determined automatically. If the age-range is found automatically, this is done using the method developed for the generalized growth-balance method. Part of this method relies on a prior value for remaining life expectancy in the open age group. By default, this is estimated using a standard reference to the Coale-Demeny West model life table, although the user may also supply a value."

GGB-SEG:

"The method estimates age-specific degrees of coverage. The age pattern of these is assumed to be noisy, so we take the arithmetic mean over some range of ages. One may either specify a particular age-range, or let the age range be determined automatically. If the age-range is found automatically, this is done using the method developed for the generalized growth-balance method. Part of this method relies on a prior value for remaining life expectancy in the open age group. By default, this is estimated using a standard reference to the Coale-Demeny West model life table, although the user may also supply a value. The difference between this method and SEG is that here we adjust census 1 part way through processing, based on some calculations similar to GGB."

Two different approaches were performed initially: 1) Estimate completeness using the age ranges automatically chosen by the R DDM package; and 2) Estimate completeness estimates using the specific age ranges for each method as described in the Murray et al. 2010 paper. Results from the three DDM methods with both age range selection strategies were obtained for men and women for all Ecuador and for the 22 study areas. Following Murray et al. (2010) recommendation, a summary measure of the three DDM methods was obtained. In that study, the authors used the median as summary measure. The problem with that approach is that the median of three numbers will always be one of the three values. In our case that meant selecting either the SEG or the GGBSEG methods most of the time. For that reason we decided to use the harmonic mean, as it is a central tendency measure not affected by extreme values and appropriate for rates.

The following table compares the completeness results obtained using the automatic age range selection with the ones obtained using the specific age ranges:

Table 1: Comparison of DDM estimates using automatic age trims of the R DDM package and age trims recommended by Murray et al. (2010)

Area	Women									Men									
	Ages DDM package R				Ages Murray et al. 2010				Mean Diferences	Ages DDM package R				Ages Murray et al. 2010				Mean Diferences
	GGB*	SEG**	GGB - SEG***	Mean	GGB*	SEG**	GGB - SEG***	Mean		GGB*	SEG**	GGB - SEG***	Mean	GGB*	SEG**	GGB - SEG***	Mean	
**Azuay**	98.82	80.50	66.91	80.03	66.08	75.21	78.60	72.90	7.13	107.10	91.49	72.69	88.17	66.45	79.45	74.57	73.09	15.08
**Bolivar**	98.81	71.91	71.29	78.83	68.94	76.64	78.11	74.34	4.49	95.56	79.52	74.28	82.18	64.93	78.69	72.73	71.67	10.51
**Cañar**	99.87	57.49	61.89	68.86	56.43	63.50	62.80	60.74	8.12	95.54	79.06	70.87	80.59	57.18	73.01	66.03	64.75	15.84
**Carchi**	80.20	56.76	55.98	62.56	58.30	62.13	66.84	62.23	0.34	85.18	60.80	62.58	67.92	66.75	67.42	76.84	70.05	-2.13
**Cotopaxi**	99.92	83.67	72.23	83.79	72.06	81.38	79.27	77.35	6.44	101.66	84.40	65.66	81.27	61.09	74.49	69.53	67.91	13.36
**Chimborazo**	96.01	78.20	74.17	81.78	67.34	77.35	78.42	74.02	7.76	95.61	93.55	78.81	88.66	66.26	82.06	77.04	74.52	14.14
**El Oro**	76.86	55.03	50.83	58.99	48.65	54.48	55.99	52.84	6.14	68.33	62.60	55.16	61.55	49.68	58.35	61.59	56.07	5.48
**Esmeraldas**	43.07	86.01	63.62	59.33	27.30	44.38	40.54	35.79	23.54	43.94	106.46	72.29	65.24	27.58	50.14	45.37	38.34	26.90
**Guayas / Santa Elena**	57.04	57.70	46.62	53.27	48.73	54.28	58.82	53.62	-0.35	54.37	67.21	51.12	56.79	48.34	57.81	61.90	55.41	1.37
**Imbabura**	101.18	86.67	73.53	85.66	72.29	82.05	82.46	78.64	7.02	100.32	86.91	74.17	85.82	73.30	83.00	83.22	79.56	6.26
**Loja**	100.81	62.41	59.31	70.09	54.93	63.90	63.81	60.58	9.51	101.55	79.32	71.79	82.45	64.16	74.96	72.53	70.24	12.21
**Los Rios**	75.27	68.77	52.78	64.14	49.50	60.57	60.29	56.29	7.85	70.13	77.06	57.89	67.40	49.14	62.98	63.04	57.59	9.81
**Manabi**	82.71	63.62	55.61	65.51	50.92	60.66	62.40	57.53	7.98	85.19	74.86	61.20	72.40	54.63	66.64	67.51	62.34	10.06
**Morona Santiago**	46.48	39.80	33.43	39.19	27.74	35.30	31.51	31.21	7.98	54.03	63.17	49.05	54.82	38.18	47.83	42.25	42.39	12.43
**Napo**	62.22	67.23	53.19	60.31	38.44	49.36	45.52	43.96	16.34	71.98	88.74	64.05	73.57	51.22	65.98	58.20	57.85	15.73
**Pastaza**	40.59	73.71	56.18	53.57	44.55	52.77	47.35	47.99	5.58	45.81	61.87	48.02	51.01	36.44	44.82	44.40	41.51	9.50
**Pichincha / Santo Domingo**	72.07	81.98	65.03	72.37	65.80	73.09	78.46	72.07	0.30	64.46	81.18	63.62	68.89	65.86	72.36	79.12	72.04	-3.16
**Tungurahua**	98.69	83.24	82.22	87.44	78.41	83.89	92.45	84.53	2.91	99.48	87.86	78.87	87.94	75.80	84.42	89.97	82.98	4.96
**Zamora Chinchipe**	53.53	45.88	39.20	45.46	41.05	44.73	41.30	42.29	3.17	47.16	52.06	41.78	46.62	33.03	42.25	40.24	38.07	8.55
**Galapagos**	12.58	35.10	30.43	21.30	16.99	18.03	19.23	18.03	3.27	25.46	45.66	38.83	34.51	44.09	41.12	46.90	43.91	-9.40
**Sucumbios**	22.00	49.52	41.57	33.45	16.79	25.44	24.66	21.52	11.93	28.53	58.70	46.24	40.70	23.08	34.82	33.59	29.46	11.24
**Orellana**	28.85	105.65	74.30	52.10	26.96	42.62	37.80	34.48	17.62	36.30	126.62	79.87	62.54	38.41	55.71	47.15	46.02	16.52
**Ecuador**	71.97	67.67	57.21	65.00	55.75	63.43	66.59	61.58	3.43	71.76	74.68	60.09	68.23	55.50	65.81	68.41	62.72	5.51

* Generalized growth balance method

** Synthetic extinct generations method

*** Hybrid generalized growth balance and synthetic extinct generations method

From the table shown above, we can see that for both sexes, completeness estimates were higher using the automatic selection of age ranges in most of the study areas. When applying DDM methods for the whole country, both strategies obtained similar results (mean difference of 3.43% for women and 5.51% for men). The difference of the harmonic means was much greater at the provincial level; ranging from -0.35% (Pichincha / Santo Domingo) to 23.54% (Esmeraldas) in women; and from -9.40% (Galápagos) to 26.90% (Esmeraldas) in men. We decided to keep the results obtained through the automatic age range selection for two reasons:

1) These estimates give more conservative estimates of completeness, closer to the ones observed in previous literature for Ecuador.
2) One of the potential benefits of using the specific age ranges approach was to reduce the bias associated with migration. We compared the changes in DDM estimates with the provincial percentages of net migration in 2001 and 2010 reported by Royuela and Ordóñez (2018) (8). The change seen between the two approaches did not seem to be related to the migratory patterns in Ecuador.

8. Results

The specific results are presented in the article. Here we present the graphical representation of the three completeness estimates and their harmonic mean for the 22 study areas in men and women:

Figure 1: DDM results for the 22 study areas - Women

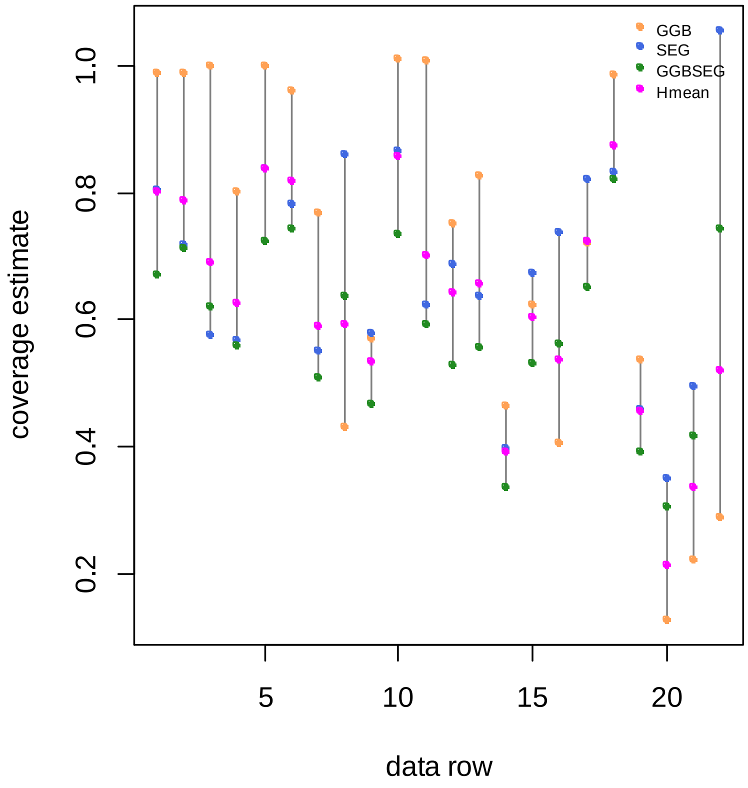

Figure 2: DDM results for the 22 study areas - Men

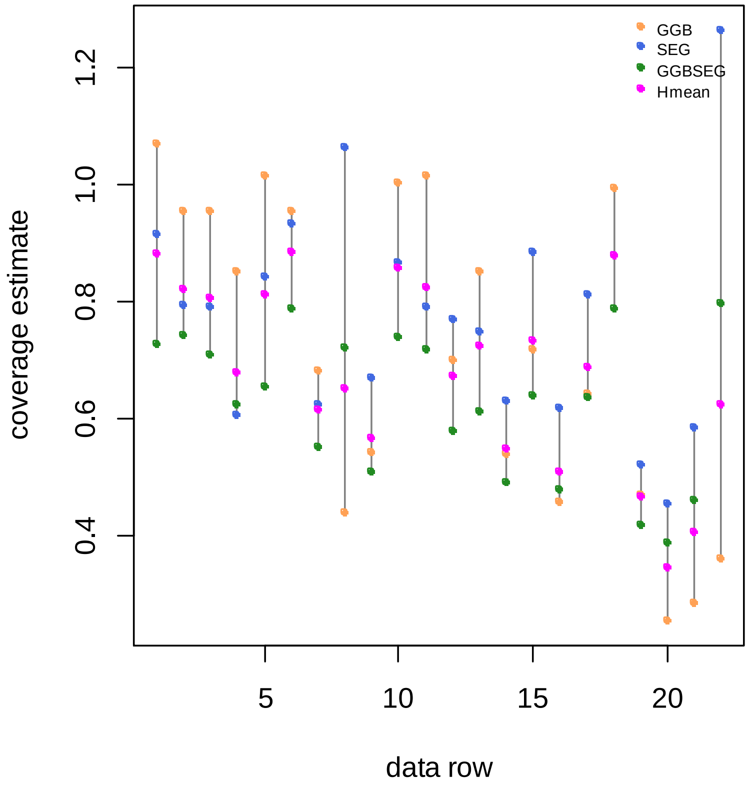

9. Bibliography

1. Hill K. Analytical Methods to Evaluate the Completeness and Quality of Death Registration: Current State of Knowledge. 2017;

2. Murray CJL, Rajaratnam JK, Marcus J, Laakso T, Lopez AD. What can we conclude from death registration? Improved methods for evaluating completeness. PLoS Med. 2010;7(4).

3. Adair T, Lopez AD. Estimating the completeness of death registration: An empirical method. Queiroz B, editor. PLoS One. 2018 May 30;13(5):e0197047.

4. Hill K, Queiroz B. Adjusting the general growth balance method for migration. Rev Bras Estud Popul. 2010;27(March):7–20.

5. Hill K, You D, Choi Y. Death distribution methods for estimating adult mortality: Sensitivity analysis with simulated data errors. Demogr Res. 2009;21:235–54.

6. Riffe T, Lima E, Queiroz B. Package “DDM”: Death Registration Coverage Estimation. 2017.

7. INEC. Evolución de las variables investigadas en los censos de población y vivienda del Ecuador 1950, 1962, 19749 1982, 1990, 2001 y 2010. Inec. 2010;

8. Royuela V, Ordóñez J. Internal migration in a developing country: A panel data analysis of Ecuador (1982-2010). Pap Reg Sci. 2016;
