# Supplementary figures and images for: Evaluation of the mortality registry in Ecuador (2001–2013) – social and geographical inequalities in completeness and quality
Source: Popul Health Metr. 2019 Mar 28;17:3. doi: 10.1186/s12963-019-0183-y (PMC6437878; doi:10.1186/s12963-019-0183-y)

# Yearly Garbage Codes % in the Study Period – Women

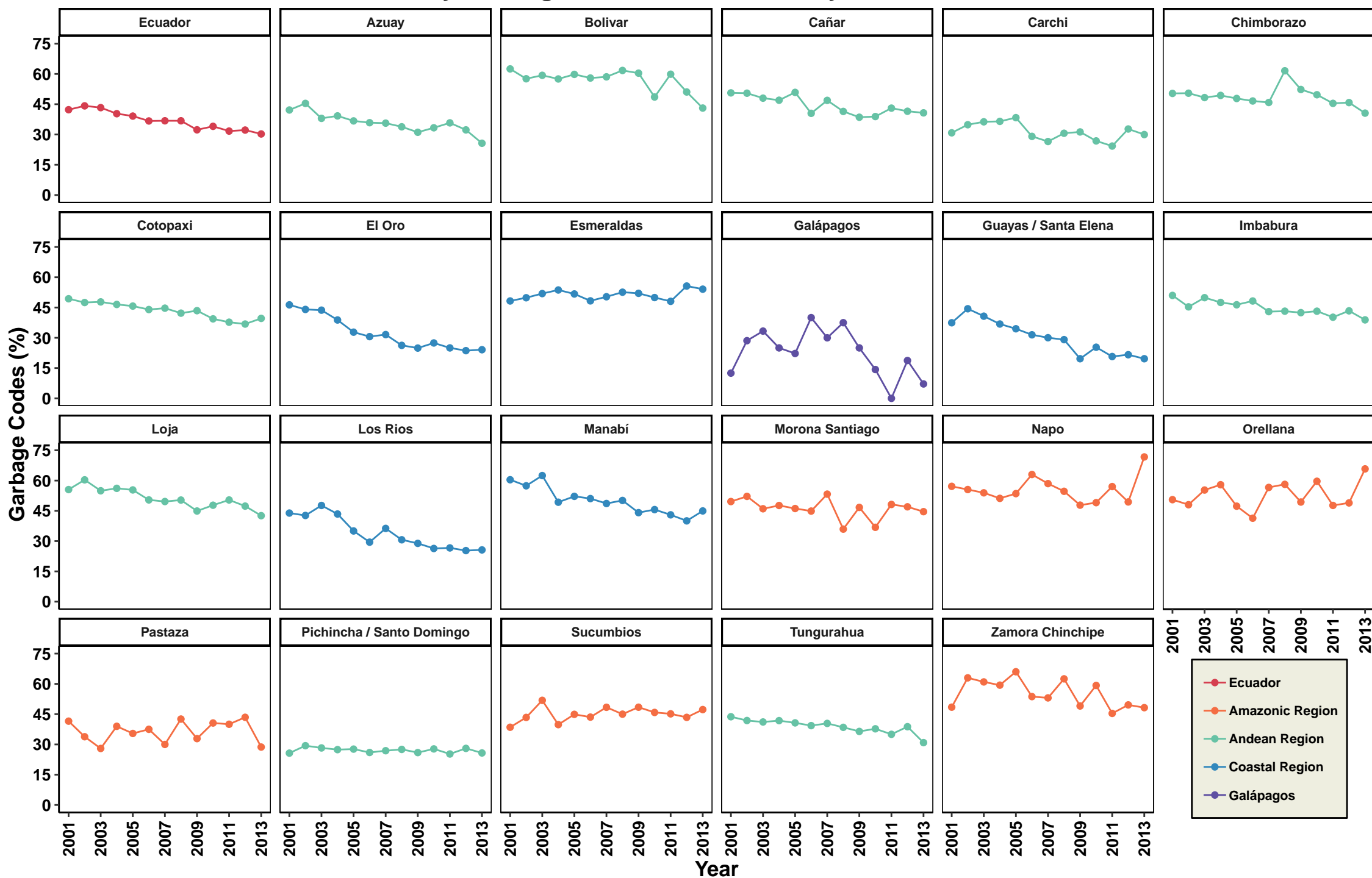

Supplement: Supplementary file 3 — : Figure S1. Evolution of garbage code percentages (2001–2013) in each of the study areas - Women (PDF 10 kb) [file 12963_2019_183_MOESM3_ESM.pdf]

# Yearly Garbage Codes (%) in the Study Period – Men

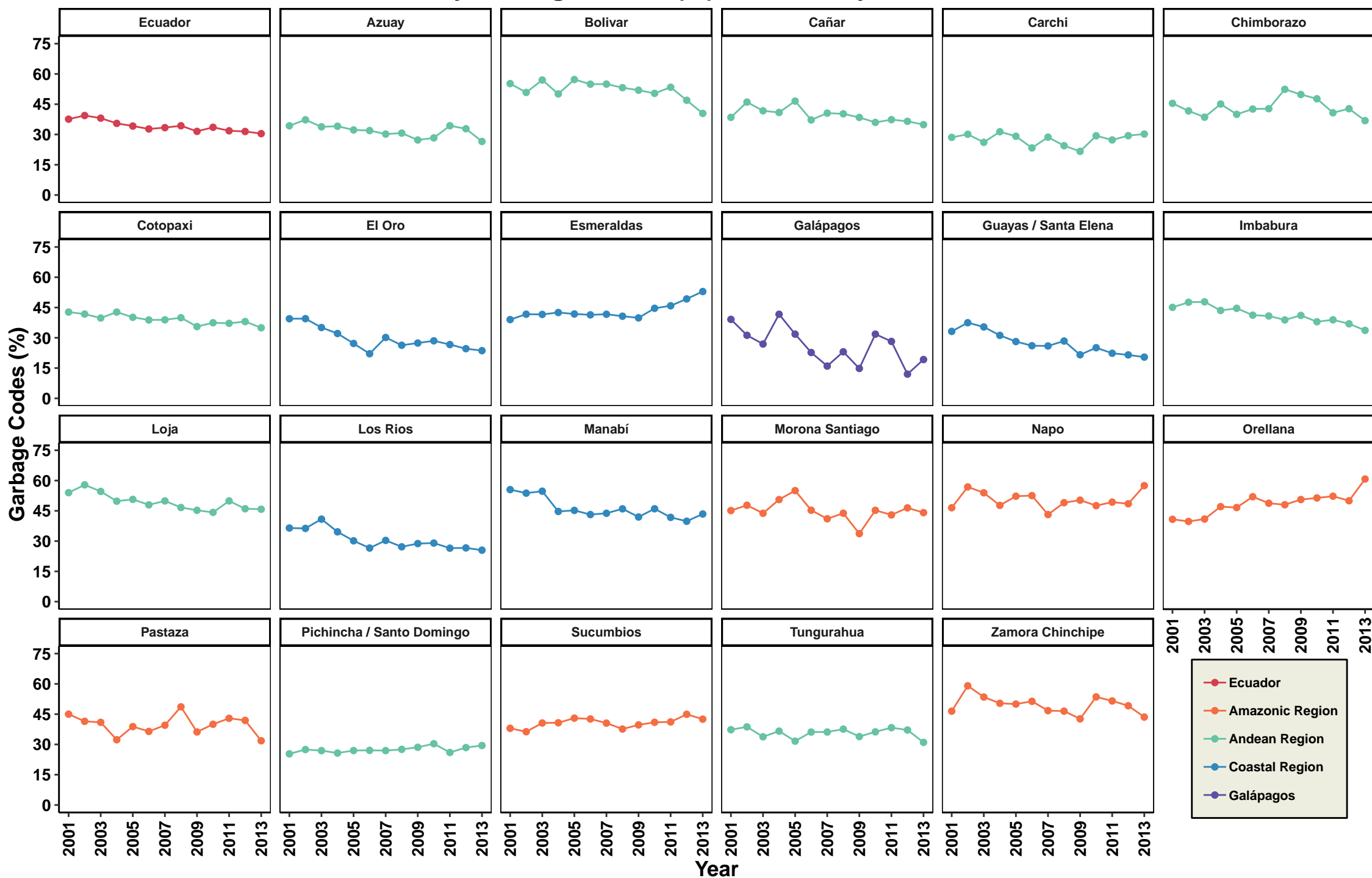

Supplement: Supplementary file 4 — : Figure S2. Evolution of garbage code percentages (2001–2013) in each of the study areas – Men (PDF 10 kb) [file 12963_2019_183_MOESM4_ESM.pdf]

# Garbage Codes and Deaths not Coded by a Medical Doctor (MD)

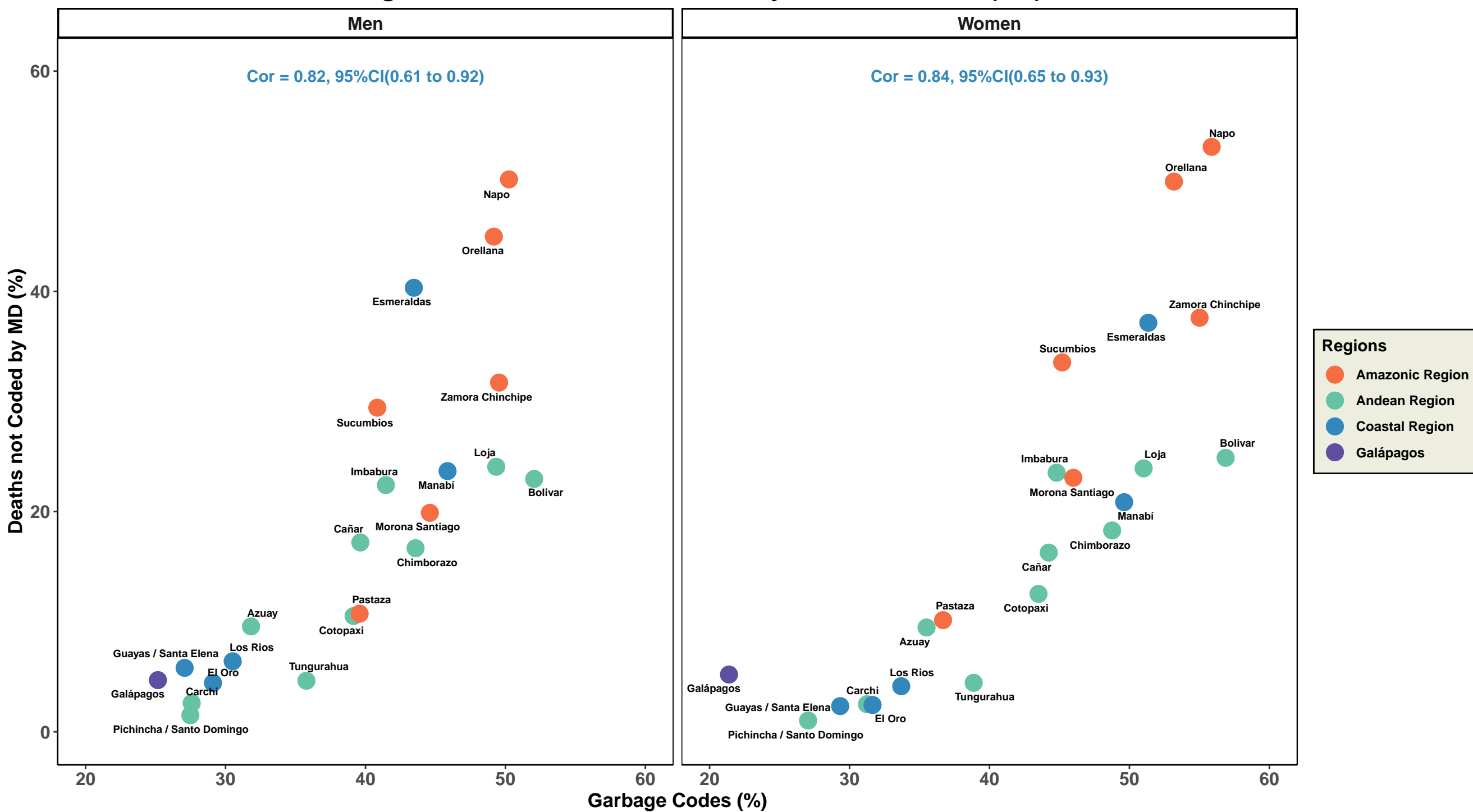

Supplement: Supplementary file 5 — : Figure S3. Garbage Code percentages and percentages of deaths not coded by a Medical Doctor (2001–2013) for Women and Men – Scatterplot (PDF 6 kb) [file 12963_2019_183_MOESM5_ESM.pdf]
